# Activating Transcription Factor 3 in Pain: A Molecular Regulator and Emerging Biomarker

**DOI:** 10.3390/genes17091034

**Published:** 2026-08-29

**Authors:** Mario García-Domínguez

**Affiliations:** Facultad de Educación, Universidad Alfonso X El Sabio (UAX), Avenida de la Universidad, 1, 28691 Villanueva de la Cañada, Spain; marigado@uax.es

**Keywords:** activating transcription factor 3, chronic pain, neuroinflammation, neuronal injury, neuroimmune signaling, axonal regeneration

## Abstract

Pain is a complex process involving dynamic transcriptional changes in cells of the PNS and CNS following injury or inflammation. Among stress-inducible transcription factors, ATF3 has emerged as one of the most robust molecular markers of neuronal injury, particularly in sensory neurons of the DRG. Although ATF3 is widely used as an indicator of axonal damage in experimental pain models, its functional contribution to the initiation, maintenance, and resolution of pain remains poorly understood. Recent transcriptomic and functional studies suggest that ATF3 not only reflects neuronal stress but also orchestrates gene expression programs involved in axonal regeneration, neuroimmune communication, ion channel remodeling, and nociceptor plasticity. Moreover, ATF3 expression has been identified in non-neuronal cell populations, including Schwann cells and satellite glial cells, indicating broader roles in peripheral nerve repair and neuroinflammation. Despite the growing body of experimental evidence, the literature remains fragmented, and no consensus has yet been reached as to whether ATF3 primarily promotes adaptive regeneration or directly contributes to maladaptive pain signaling. This review aims to provide a comprehensive and critical overview of the current understanding of ATF3 biology in pain, building on evidence from transcriptomic and molecular analyses, experimental models of neuropathic, inflammatory, and cancer-associated pain, and emerging mechanistic insights into its role in pain-related neuronal plasticity. This review examines the regulation of ATF3 expression, its downstream transcriptional targets, its interactions with inflammatory signaling pathways, and its potential value as a therapeutic target. By consolidating current evidence and highlighting existing knowledge gaps, this review seeks to clarify the multifaceted role of ATF3 in pain pathophysiology.

## 1. Introduction

Pain is one of the most prevalent and debilitating health conditions worldwide, representing a major medical, social, and economic challenge. As defined by the International Association for the Study of Pain (IASP), pain is “an unpleasant sensory and emotional experience associated with, or resembling that associated with, actual or potential tissue damage” [1,2]. Beyond its physiological role as a protective warning signal, persistent pain becomes a pathological state that affects physical function, psychological well-being, and quality of life [3]. Chronic pain is currently recognized as a disease in its own right rather than merely a symptom of underlying pathology, reflecting the complex neurobiological alterations that sustain pain long after the initial injury has resolved. The global burden of chronic pain has increased steadily over recent decades, driven by population aging, the growing prevalence of metabolic and degenerative diseases, cancer survivorship, and improved life expectancy [4,5]. Epidemiological data suggest that approximately 20–30% of adults worldwide experience chronic pain, with significant variation depending on diagnostic criteria, geographical region, and population characteristics [6,7].

The transition from acute to chronic pain is recognized as a dynamic biological process driven by peripheral and central sensitization, which induce plastic changes throughout the PNS and CNS [8,9]. Peripheral sensitization develops following tissue injury or inflammation through increased responsiveness of primary sensory neurons to chemical, mechanical, or thermal stimuli [10]. Persistent nociceptive input from sensitized peripheral afferents subsequently triggers central sensitization within the dorsal horn and supraspinal pain-processing circuits, leading to long-lasting increases in neuronal excitability, synaptic efficacy, and network reorganization [11].

A growing body of evidence demonstrates that these alterations are underpinned by extensive changes in gene expression. Following nerve injury (or inflammation), nociceptors rapidly activate transcriptional programs that regulate cellular stress responses, axonal regeneration, metabolic adaptation, neurotransmitter synthesis, ion channel expression, and inflammatory signaling [12]. Similar transcriptional remodeling also develops in spinal cord neurons, glial cells, Schwann cells, macrophages, and infiltrating immune cells, highlighting that chronic pain is the consequence of multicellular interactions rather than neuron-autonomous dysfunction alone [13,14].

Among the molecular regulators governing these transcriptional responses, stress-responsive transcription factors occupy a key position. Members of several transcription factor families, like c-Jun, STAT3, NF-κB, Sox11, KLFs, CREB, and the activating transcription factor (ATF) family, are promptly induced after neuronal injury, where they orchestrate transcriptional programs governing survival, inflammation, regeneration, and plasticity [15,16]. These transcription factors integrate several extracellular signals generated by axonal damage, pro-inflammatory mediators, oxidative stress, and endoplasmic reticulum stress into coordinated transcriptional responses that ultimately determine cellular fate [17]. Among these injury-responsive transcription factors, ATF3 has emerged as one of the strongest and most reproducible molecular markers of neuronal stress [18]. ATF3 is part of ATF/cAMP response element-binding (ATF/CREB) family of basic leucine zipper (bZIP) transcription factors and is considered an immediate early response gene whose expression is rapidly induced by diverse cellular stressors [19]. Under physiological conditions, ATF3 expression is extremely low or undetectable in most adult sensory neurons. Following peripheral nerve injury, however, ATF3 is upregulated in DRG neurons within hours of axonal damage, making it one of the most widely employed biomarkers for identifying injured neurons in several experimental models of neuropathic pain [20,21]. Similar induction has been observed after spinal cord injury (SCI), traumatic brain injury (TBI), chemotherapy-induced neuropathy (CIPN), peripheral nerve transection and compression, and other inflammatory conditions, underscoring its role as a highly conserved component of the neuronal stress response [22,23,24,25].

Although ATF3 has traditionally been regarded as a surrogate marker of axonal injury, accumulating evidence indicates that its biological functions extend far beyond injury detection. ATF3 acts as a context-dependent transcriptional regulator capable of both activating and repressing gene expression with other members of the AP-1 and ATF/CREB transcription factor families [26,27]. Through these interactions, ATF3 influences some biological processes, like axonal regeneration, neuronal survival, cytoskeletal remodeling, mitochondrial homeostasis, metabolic adaptation, autophagy, apoptosis, immune regulation, and cell differentiation [28,29]. Genome-wide transcriptomic and chromatin accessibility studies increasingly suggest that ATF3 functions as a master regulator of injury-induced gene networks rather than simply reflecting neuronal damage [30,31].

Despite its well-established role as an injury marker, the relationship between ATF3 and pain remains unclear. Experimental studies have demonstrated robust ATF3 induction in virtually all major rodent models of neuropathic pain, including spinal nerve ligation (SNL), chronic constriction injury (CCI), spared nerve injury (SNI), sciatic nerve transection (SNT), diabetic neuropathy (DPN), and CIPN [32,33,34,35,36,37]. Consistent with these observations, some studies have shown that ATF3 regulates genes involved in ion channel remodeling, neuropeptide synthesis, macrophage recruitment, and neuroimmune communication, processes that are central to peripheral sensitization and chronic pain [18,38]. In inflammatory pain models, ATF3 induction is more heterogeneous and appears to depend on the intensity, duration, and anatomical location of the inflammatory insult [39,40].

An additional layer of complexity has emerged with the recognition that ATF3 is not restricted to neurons. Recent studies have shown injury-induced ATF3 expression in microglia, Schwann cells, satellite glial cells, macrophages, and other non-neuronal populations involved in peripheral nerve repair [18,41]. In Schwann cells, ATF3 orchestrates the transition toward a regenerative phenotype that enhances axonal regrowth and remyelination [42]. Within DRG, satellite glial cells expressing ATF3 might influence inflammatory signaling and neuron-glia communication, whereas immune cell-specific ATF3 modulates cytokine synthesis and contributes to the resolution of inflammatory responses [43]. Collectively, these findings suggest that ATF3 orchestrates multicellular responses within the injured nervous system, linking neuronal stress, immune activation, and tissue repair.

This review provides a comprehensive overview of the current understanding of the molecular regulation of ATF3 expression, its downstream transcriptional programs, and its interplay with inflammatory and stress-responsive signaling pathways. It further integrates evidence from transcriptomic studies and experimental models of neuropathic and inflammatory pain, with particular emphasis on the emerging functions of ATF3 in both neuronal and non-neuronal cell populations. In addition, this review discusses the translational implications of modulating ATF3-dependent pathways as a potential therapeutic strategy for chronic pain and identifies crucial knowledge gaps and unresolved questions that should guide future investigations in this rapidly advancing field.

## 2. ATF3: Biology and Regulation

### 2.1. Molecular Biology of ATF3

Activating Transcription Factor 3 is a stress-inducible transcription factor belonging to the ATF/CREB family of bZIP proteins, which is a member of the AP-1 superfamily of transcriptional regulators [19]. Unlike constitutively expressed transcription factors, ATF3 functions as an immediate-early response gene whose expression is rapidly induced by a wide variety of physiological and pathological stimuli, such as oxidative stress, ER stress, DNA damage, pro-inflammatory cytokines, nutrient deprivation, metabolic stress, ischemia, hypoxia, and mechanical injury [44]. This induction enables ATF3 to act as a central molecular integrator of diverse intracellular signaling pathways, coordinating transcriptional programs that orchestrate cell survival, proliferation, differentiation, apoptosis, immune responses, and tissue repair [38].

**Figure 1:**
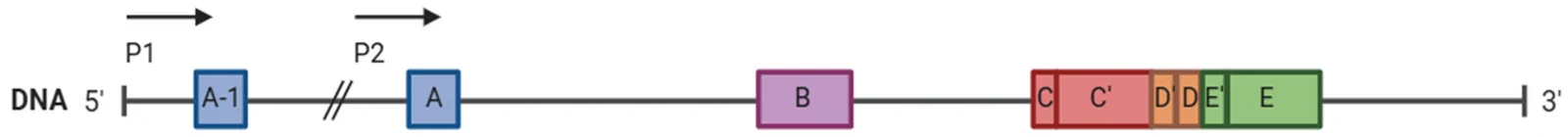
Schematic representation of the human ATF3 gene organization. The human ATF3 locus spans 56 kb on chromosome 1q32.3 and comprises five exons (A, B, C, D, and E). Full-length ATF3 transcripts are generated from two distinct promoters, P1 and P2, and differ exclusively in their 5′ UTRs through the incorporation of either exon A-1 or exon A, respectively. P1 is a non-canonical promoter located approximately 43.5 kb upstream of P2. Alternative splicing of the primary transcript, via the use of distinct splice donor and acceptor sites, gives rise to multiple ATF3 transcript variants with different exon compositions and transcript lengths.

The human ATF3 gene (Figure 1), placed on chromosome 1q32.3, encodes a relatively small protein composed of 181 amino acids, with a molecular weight of approximately 22 kDa [45]. Under physiological conditions, ATF3 expression is low or undetectable in most tissues as a result of its rapid turnover and strict transcriptional regulation [46]. Following cellular stress, however, ATF3 mRNA and protein levels increase rapidly within minutes, highlighting its key role as an immediate-early transcriptional regulator [47]. The protein is localized in the nucleus, where it modulates gene expression through direct interaction with specific DNA sequences, although transient cytoplasmic localization has also been reported under certain stress conditions [48].

The biological activity of ATF3 is determined by its bZIP structure, which consists of two highly conserved functional domains [49]. The N-terminal region is intrinsically disordered and serves as a regulatory platform rather than possessing intrinsic catalytic activity. This flexible domain enables ATF3 to interact with a wide variety of transcriptional co-regulators, chromatin-remodeling proteins, and signaling molecules [50]. Moreover, it contains multiple residues that undergo post-translational modifications, including phosphorylation, acetylation, ubiquitination, and SUMOylation, which regulate protein stability, transcriptional activity, and subcellular localization [26,51]. Through this intrinsically disordered region, ATF3 can recruit either transcriptional co-activators, including p300, or transcriptional co-repressors like histone deacetylases (HDACs; HDAC1 and HDAC2), Nuclear Receptor Co-Repressor (NCoR), and Silencing Mediator for Retinoid and Thyroid Hormone Receptors (SMRT), thus functioning as a transcriptional activator or repressor depending on the cellular context [52,53,54].

Adjacent to the regulatory N-terminal region lies the strongly conserved basic DNA-binding domain, which is enriched in positively charged lysine and arginine residues that interact with the negatively charged DNA backbone [55]. This domain recognizes specific cis-regulatory sequences, primarily the cAMP response element (CRE; 5′-TGACGTCA-3′) and related ATF/AP-1 consensus motifs located within the promoters of target genes [56]. Structural studies have demonstrated that the α-helices of the basic region insert into the major groove of DNA, allowing sequence-specific recognition through hydrogen bonding and hydrophobic interactions with nucleotide bases [57,58].

The C-terminal leucine zipper domain mediates protein dimerization and represents the defining structural feature of the bZIP family [59]. This domain encompasses leucine residues positioned every seventh amino acid, creating an amphipathic α-helix that forms stable coiled-coil structures through hydrophobic interactions with another bZIP protein [60]. ATF3 can therefore form either homodimers or heterodimers with some members of the AP-1 family, like c-Jun, JunB, JunD, ATF2, ATF4, CHOP (DDIT3), and C/EBP [26].

Finally, the identity of the dimerization partner governs the transcriptional outcome. ATF3 homodimers generally serve as transcriptional repressors by recruiting HDAC-containing repressor complexes that induce chromatin condensation and inhibit gene expression [61]. In contrast, heterodimers with transcriptionally active partners like c-Jun or ATF2 recruit CBP/p300 histone acetyltransferases, causing histone acetylation, chromatin relaxation, and activation of transcription [62,63]. Accordingly, ATF3 operates as a context-dependent molecular switch whose activity is regulated by dimer composition, chromatin accessibility, and the surrounding signaling environment [38].

### 2.2. Regulation of ATF3 Expression

Transcriptional activation of ATF3 is regulated through its promoter, which contains several stress-responsive cis-regulatory elements recognized by transcription factors activated under different cellular conditions [64]. Among these are CREs, AP-1 binding sites, NF-κB response elements, C/EBP motifs, and binding sites for ATF4, CHOP (DDIT3), and XBP1 [44,65]. A major mechanism governing ATF3 expression is the Integrated Stress Response (ISR), which is activated by numerous cellular insults [66]. During ER stress, amino acid deprivation, oxidative stress, viral infection, or heme deficiency, the stress-responsive kinases PERK (EIF2AK3), GCN2 (EIF2AK4), PKR (EIF2AK2), and HRI (EIF2AK1), respectively, phosphorylate the translation initiation factor eIF2α [67]. Although phosphorylation of eIF2α inhibits general protein synthesis, it simultaneously facilitates the selective translation of ATF4, which transcriptionally activates ATF3 through direct promoter binding [68].

The unfolded protein response (UPR), activated by the accumulation of misfolded proteins in the ER, also plays a major role in ATF3 regulation [69]. While the PERK-eIF2α–ATF4 axis constitutes the principal mechanism responsible for ATF3 induction during ER stress, the IRE1α-XBP1 and ATF6 signaling branches further enhance ATF3 expression by cooperating with ATF4 and CHOP to activate stress-responsive promoters [70]. Additionally, oxidative stress stimulates some MAPK pathways, including c-Jun N-terminal kinase (JNK), p38 MAPK, and extracellular signal-regulated kinase (ERK), which activate transcription factors like c-Jun, ATF2, and CREB that directly bind the ATF3 promoter [44,47]. Similarly, pro-inflammatory stimuli trigger rapid ATF3 induction via TLR-mediated activation of NF-κB and MAPK signaling [71]. Interestingly, ATF3 subsequently acts as a negative feedback regulator of inflammation by suppressing the expression of pro-inflammatory cytokines including IL-6, IL-12, and TNF-α, thus preventing excessive immune activation [72,73].

DNA damage also induces ATF3 through activation of the ATM/ATR-p53 signaling axis [74]. In turn, ATF3 modulates p53 stability and transcriptional activity by preventing its MDM2-mediated ubiquitination, thereby establishing a reciprocal regulatory loop that influences cell cycle arrest, DNA repair, senescence, and apoptosis [75]. Moreover, metabolic stress caused by nutrient deprivation, mitochondrial dysfunction, lipid overload, or reactive oxygen species (ROS) activates the ISR and AMPK-dependent pathways, leading to increased ATF3 expression in metabolically active tissues such as liver, adipose tissue, skeletal muscle, and pancreatic β-cells [29,38,76].

In addition to transcriptional regulation, ATF3 expression is modulated by epigenetic mechanisms that influence chromatin accessibility and transcriptional competence. Histone acetylation mediated by the histone acetyltransferases CBP and p300 promotes chromatin relaxation and facilitates the recruitment of RNA polymerase II to the ATF3 promoter, whereas HDACs suppress ATF3 transcription by maintaining chromatin in a condensed state [77,78]. Histone methylation also contributes to ATF3 regulation, with active chromatin marks (H3K4me3) being associated with transcriptional activation, whereas repressive marks including H3K27me3 are associated with transcriptional silencing under basal conditions [79,80,81]. In some pathological contexts, particularly in certain human cancers, CpG island hypermethylation within the ATF3 promoter has been linked to reduced ATF3 transcription, suggesting that epigenetic silencing might contribute to the loss of its tumor-suppressive functions [82].

Other regulatory complexity arises at the post-transcriptional level. ATF3 mRNA stability is controlled by numerous RNA-binding proteins and microRNAs that interact with its 3′UTR [83]. Some microRNAs, including miR-27a-3p and miR-494, have been reported to reduce ATF3 expression by promoting mRNA degradation or inhibiting translation in a tissue- and disease-specific manner [84,85]. Conversely, RNA-binding proteins like HuR (ELAVL1) stabilize ATF3 transcripts during stress responses, thus prolonging ATF3 expression when required [86]. Moreover, alternative splicing expands ATF3 functional diversity through the generation of isoforms with distinct transcriptional properties, including variants capable of acting as dominant-negative regulators through altered dimerization or DNA-binding capacity [87,88].

Finally, ATF3 protein abundance and activity are further regulated by post-translational modifications. Phosphorylation by some stress-activated kinases (e.g., JNK and p38 MAPK) regulates its transcriptional activity, DNA-binding affinity, and interactions with other transcription factors [47,89]. Acetylation mediated by CBP/p300 generally enhances ATF3 transcriptional activity by promoting chromatin association and coactivator recruitment [62,63]. In contrast, ubiquitination targets ATF3 for proteasomal degradation, resulting in a relatively short protein half-life that enables rapid termination of stress-induced transcriptional responses once cellular homeostasis has been restored [90].

## 3. ATF3 in Pain: From Injury Marker to Functional Regulator

### 3.1. ATF3 as a Marker of Neuronal Injury

Following injury, neuronal ATF3 is rapidly upregulated as part of the integrated cellular stress response, making it a fundamental indicator of damage [22,23,24,25,91]. Axonal injury drives Ca^2+^ influx, oxidative stress, mitochondrial dysfunction, and activation of several stress-responsive kinases, leading to phosphorylation of several transcription factors (e.g., c-Jun and ATF2), which subsequently promote ATF3 gene transcription [92]. Simultaneously, ER stress activates the PERK-eIF2α-ATF4 signaling cascade, further enhancing ATF3 expression as part of the UPR [70].

Once induced, ATF3 regulates the transcription of several genes involved in cell survival, apoptosis, axonal regeneration, and cytoskeletal remodeling [93]. Importantly, the functional consequences of ATF3 activation are context-dependent and are influenced by the identity of the ATF3-expressing cell, the time required for induction, and the transcriptional programs associated. The downstream effect of ATF3 activation is context-dependent, shaped by the balance between adaptive and maladaptive stress responses [38]. Thus, ATF3 induction in injured neurons should be distinguished from ATF3 activity in non-neuronal cells, while transient activation during the acute injury response should be distinguished from persistent activation associated with chronic neuronal stress.

In peripheral neurons, prolonged ATF3 expression is generally associated with a pro-regenerative transcriptional program, encouraging neurite outgrowth and axonal regeneration through the induction of regeneration-associated genes (RAGs), including *GAP43*, *SPRR1A*, and *HSP27* [94,95,96]. In this setting, neuronal ATF3 activity can therefore promote an adaptive transcriptional state characterized by axonal growth, cytoskeletal remodeling, and enhanced regenerative competence [41]. in the CNS, where the regenerative capacity is limited, ATF3 induction reflects neuronal stress and injury, although experimental overexpression has been demonstrated to enhance intrinsic regenerative competence under certain conditions [18,20,41]. Following peripheral nerve injury, nearly all damaged DRG neurons demonstrates robust nuclear ATF3 immunoreactivity after 24–48 h, whereas uninjured neurons remain ATF3-negative [97,98]. Similar induction has been reported after SCI, TBI, optic nerve crush, ischemic stroke, and neurodegenerative conditions associated with chronic cellular stress [22,23,24,25,99]. Interestingly, ATF3 expression is tightly linked to axonal damage rather than neuronal death, allowing the identification of neurons that have mounted an active injury response before irreversible degeneration occurs [96].

Beyond its value as a biomarker, accumulating evidence suggests that ATF3 actively modulates the transcriptional reprogramming required for neuronal adaptation to injury. Genome-wide studies have demonstrated that ATF3 cooperates with other regeneration-associated transcription factors, including c-Jun, STAT3, KLF family members, and Sox11, to orchestrate gene networks that modulate axonal growth, metabolic adaptation, and inflammatory signaling [100,101].

### 3.2. ATF3 in Axonal Regeneration and Neuronal Repair

ATF3 is one of the earliest and most robustly induced regeneration-associated transcription factors (RATFs) following neuronal injury [102]. Among these are GAP43, a critical mediator of growth cone formation and axonal elongation [103]; SPRR1A, which enhances neurite extension by stabilizing the actin cytoskeleton [104]; CAP-23, involved in membrane expansion and cytoskeletal plasticity [105]; and arginase-1 (Arg1), which increases polyamine synthesis necessary for microtubule assembly and axonal growth [106]. ATF3 also induces the expression of neurotrophic factor receptors, including GDNF family receptor α1 (GFRα1) and improves responsiveness to nerve growth factor (NGF), brain-derived neurotrophic factor (BDNF), and glial cell line-derived neurotrophic factor (GDNF), thereby increasing the regenerative competence of injured neurons [107,108,109,110].

Beyond its direct regulation of growth-associated genes, ATF3 orchestrates extensive transcriptional reprogramming that supports neuronal repair [111]. It promotes metabolic adaptation by activating those genes involved in glucose metabolism, mitochondrial function, and antioxidant defense [112,113]. These metabolic changes are fundamental because successful regeneration requires substantial biosynthetic capacity for membrane production, cytoskeletal assembly, and organelle transport [114]. ATF3 further contributes to regeneration by modulating cytoskeletal dynamics [20]. Through regulation of actin-binding proteins and microtubule-associated proteins, ATF3 enables growth cone formation and directional axonal extension [115]. On the other hand, increased expression of microtubule-stabilizing proteins supports elongation of the regenerating axon [116].

Growing evidence indicates that ATF3 functions cooperatively with other RATFs rather than acting alone. ATF3 forms part of a transcriptional network that comprises c-Jun, STAT3, Sox11, Smad1, KLF6, KLF7, and CREB [117,118]. There is evidence that ATF3 and STAT3 are co-induced after peripheral nerve injury and belong to the same regeneration-associated transcription factor network, but direct experimental evidence demonstrating a synergistic interaction between ATF3 and STAT3 is limited [119]. Conversely, transcription factors such as KLF4 and PTEN-mediated signaling antagonize this regenerative program by suppressing intrinsic growth capacity [120]. Therefore, the regenerative outcome depends on the balance between transcription factors that promote axon growth and those that suppress the intrinsic regenerative program, such as KLF4, together with other inhibitory signaling pathways [117,118].

One of the most remarkable features of ATF3 is the striking difference in its expression between the PNS and the CNS. Following peripheral nerve injury, DRG neurons and motor neurons exhibit rapid and sustained ATF3 induction, initiating a robust regenerative response that supports axonal regrowth and functional recovery [20,97]. Conversely, CNS neurons generally display weaker ATF3 expression after injury [18,41]. This limited ATF3 induction, together with the decreased growth competence of mature CNS neurons and the inhibitory extracellular environment (characterized by myelin-associated inhibitors, chondroitin sulfate proteoglycans -CSPGs-, and persistent neuroinflammation) contributes to the poor regenerative capacity of the adult CNS [121].

Experimental overexpression studies have demonstrated that ATF3 enhances axonal regeneration in multiple injury models. Viral-mediated ATF3 expression in sensory neurons fosters neurite elongation and accelerates peripheral nerve regeneration [122]. Similarly, transgenic overexpression of ATF3 further enhances nerve regeneration when combined with complementary pro-regenerative strategies, like PTEN deletion or activation of the mTOR signaling pathway [123,124]. These findings illustrate that ATF3 alone is insufficient to overcome the inhibitory CNS environment but significantly potentiates regeneration when integrated into combined therapeutic strategies targeting both intrinsic neuronal growth programs and extrinsic inhibitory mechanisms.

In addition to its direct neuronal functions, ATF3 influences the injury microenvironment by regulating inflammatory signaling. Depending on the cellular context, ATF3 negatively regulates TLR-dependent NF-κB activation, limiting excessive production of pro-inflammatory cytokines like TNF-α, IL-1β, and IL-6 [71]. This immunomodulatory function might reduce secondary neuronal damage and create a more permissive environment for regeneration [125]. During the acute response to injury, ATF3 induction represents an adaptive component of the cellular stress response, contributing to transcriptional reprogramming, inflammatory control, and upregulation of regenerative pathways [111]. However, long-lasting ATF3 activation might also contribute to maladaptive plasticity, neuropathic pain, or chronic stress responses, highlighting that the temporal dynamics of ATF3 expression are critical for determining its beneficial versus detrimental effects [126]. Importantly, extended expression should not be considered inherently maladaptive, because sustained ATF3 expression in peripheral neurons can also support regeneration. Rather, its consequences appear to depend on whether sustained ATF3 activity remains coupled to regenerative programs or becomes associated with persistent nociceptive and maladaptive plasticity programs [21].

### 3.3. ATF3 in Neuroimmune Signaling

Neuroimmune interactions are essential to the initiation and persistence of chronic pain, particularly following peripheral nerve injury [127]. Tissue damage initiates a highly coordinated response involving injured nociceptors, Schwann cells, satellite glial cells, infiltrating macrophages, and spinal glial cells, which collectively establish a pro-inflammatory microenvironment that potentiates peripheral and central sensitization [128]. Within this complex cellular network, ATF3 has emerged as a pivotal stress-responsive transcription factor linking neuronal injury to immune activation [129]. Although ATF3 has long been regarded as one of the most reliable molecular markers of axotomized DRG neurons, accumulating evidence indicates that it actively regulates neuroimmune communication by governing pro-inflammatory signaling, transcriptional plasticity, and regenerative responses that ultimately shape pain outcomes [130].

Peripheral nerve injury triggers ATF3 expression through the convergence of multiple intracellular stress pathways. Mechanical disruption of the axon generates membrane depolarization and sustained Ca^2+^ influx through VGCCs and TRPs, leading to intracellular Ca^2+^ overload [131,132]. Elevated cytosolic Ca^2+^ concentrations stimulate the activation of CaMKs, calcineurin, and PKC while concurrently perturbing mitochondrial Ca^2+^ homeostasis [133]. Mitochondrial dysfunction produces excessive production of ROS, ATP depletion, and loss of mitochondrial membrane potential, all of which activate apoptosis signal-regulating kinase 1 (ASK1), an upstream regulator of stress-activated MAPK signaling [134]. ASK1 phosphorylates MKK4/7 and MKK3/6, resulting in activation of JNK and p38 MAPK, respectively [135]. JNK phosphorylates c-Jun at Ser63 and Ser73, whereas p38 activates ATF2 and CREB, facilitating their binding to CRE and AP-1 motifs within the ATF3 promoter [136]. The PERK-eIF2α pathway selectively stimulates the translation of ATF4, which directly stimulates ATF3 transcription [70]. Additional input from the integrated stress response (ISR), triggered by oxidative stress, amino acid deprivation, hypoxia, and mitochondrial dysfunction, further amplifies ATF3 expression, enabling it to function as an early molecular sensor of neuronal damage [137].

**Figure 2:**
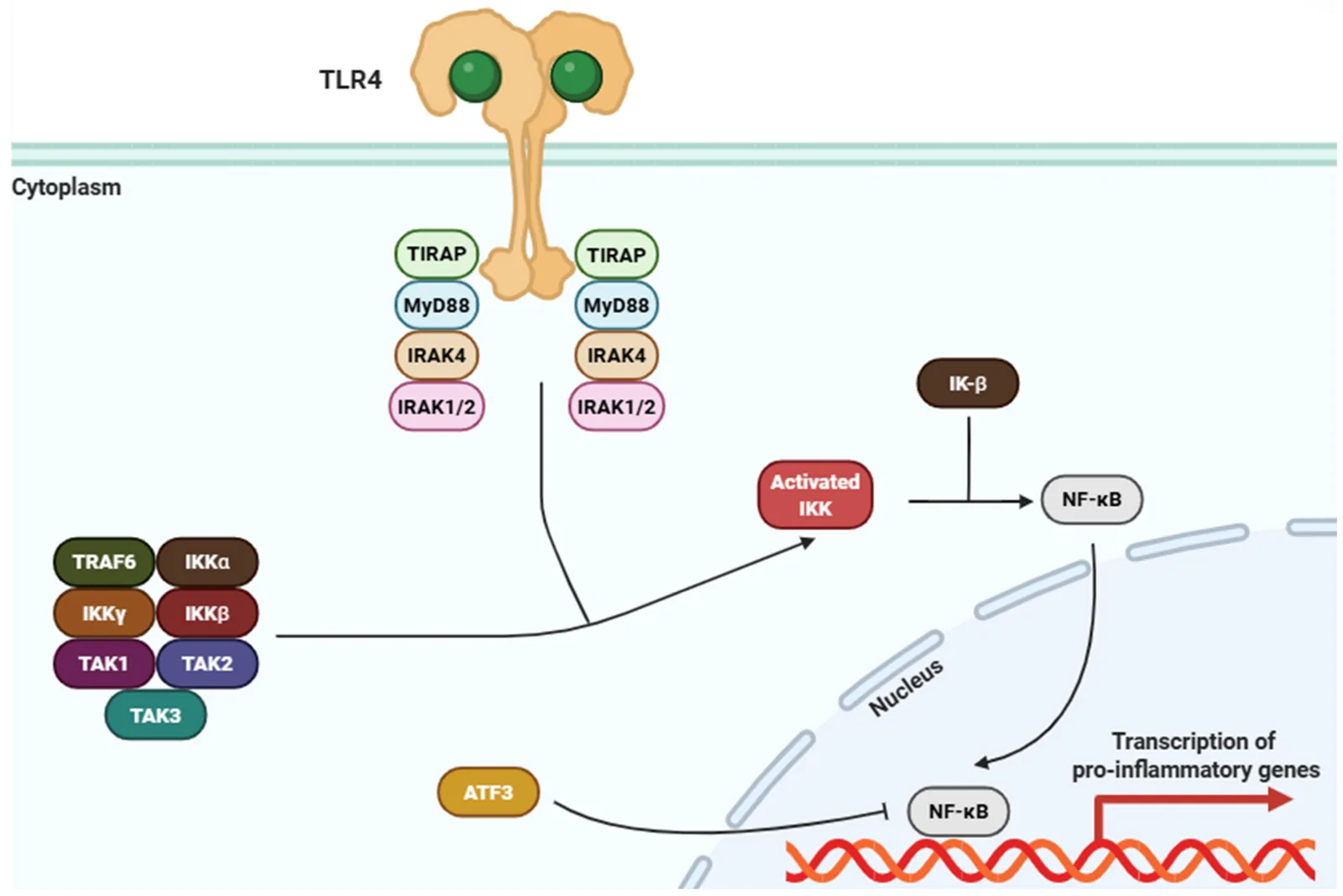
The figure illustrates the MyD88-dependent TLR4 signaling pathway, which activates the transcription factor NF-κB and promotes the expression of pro-inflammatory genes. Upon activation, TLR4 recruits the adaptor proteins TIRAP and MyD88, leading to the activation of IRAK4 and IRAK1/2. This signaling cascade activates TRAF6 and the TAK complex, which in turn stimulates the IKK complex. Activated IKK phosphorylates IκB, causing its degradation and allowing NF-κB to translocate into the nucleus, where it induces the transcription of pro-inflammatory genes. ATF3 is shown as a negative regulator that prevents NF-κB-dependent transcription, contributing to the control of the inflammatory response. Abbreviations: TLR4 (Toll-like receptor 4); TIRAP (Toll/interleukin-1 receptor domain-containing adaptor protein); MyD88 (myeloid differentiation primary response protein 88); IRAK4 (interleukin-1 receptor-associated kinase 4); IRAK1/2 (interleukin-1 receptor-associated kinases 1 and 2); TRAF6 (tumor necrosis factor receptor-associated factor 6); TAK1 (transforming growth factor-β-activated kinase 1); TAK2 (TAK1-binding protein 1); TAK3 (TAK1-binding protein 2); IKK (IκB kinase complex); IKKα (IκB kinase alpha); IKKβ (IκB kinase beta); IKKγ (IκB kinase gamma); IκB (inhibitor of nuclear factor kappa B); NF-κB (nuclear factor kappa-light-chain-enhancer of activated B cells); and ATF3 (activating transcription factor 3).

Beyond its induction in injured neurons, ATF3 occupies a central position within the communication between nociceptors and immune cells [40]. Damaged nociceptors release DAMPs, ATP, HMGB1, CSF1, CCL2, and CX3CL1 that recruit macrophages, satellite glial cells, Schwann cells, neutrophils, and dendritic cells [12,138,139]. These mediators bind to TLRs (e.g., TLR4), RAGE, purinergic receptors (e.g., P2X4), CSF1R, CCR2, and CX3CR1 expressed by immune cells (Figure 2) [140,141]. The activation of these receptors allows MyD88- and TRIF-dependent signaling cascades that converge on IKK, NF-κB, ERK, JNK, and p38 MAPK pathways, resulting in robust production of inflammatory mediators including TNF-α, IL-1β, IL-6, PGE_2_, NO, ROS, CCL2, CXCL1, and GM-CSF [142,143]. These mediators evoke the sensitization of nociceptors by increasing the expression and/or activity of VGSCs (e.g., Nav1.7, Nav1.8, and Nav1.9), TRPV1, TRPA1, ASICs, and P2X3 receptors [144]. Simultaneously, pro-inflammatory signaling enhances phosphorylation of ion channels via PKA, PKC, and MAPKs, lowering activation thresholds and increasing spontaneous firing of primary afferents [145,146].

ATF3 functions as a negative feedback regulator within this inflammatory network. After activation by TLR and MAPK signaling, ATF3 translocates to the nucleus, where it binds CRE and AP-1 regulatory elements within promoters of several pro-inflammatory genes [48]. Depending on the cellular context and dimerization partner, ATF3 may function as either a transcriptional activator or repressor; however, in macrophages and other innate immune cells, its principal role is transcriptional repression. This distinction is particularly relevant to the apparently opposing effects of ATF3 after nerve injury. In injured neurons, ATF3 is linked to transcriptional programs regulating axonal regeneration [94,95,96] and, in nociceptors, sensory excitability [111]. However, in non-neuronal cells such as macrophages, ATF3 functions as a regulator of inflammatory transcription [72,73]. Thus, neuronal and non-neuronal ATF3 should not be considered functionally equivalent, even though both are induced as part of the injury response.

Conversely, ATF3 recruits HDAC1, SIN3A-containing co-repressor complexes, and other chromatin-remodeling proteins that decrease histone H3 and H4 acetylation, resulting in chromatin condensation and reduced transcriptional accessibility [147]. As a consequence, transcription of TNF-α, IL-6, IL-12, PTGS2, CCL4, and other pro-inflammatory genes is suppressed [72]. ATF3 interferes with NF-κB signaling by decreasing p65 transcriptional activity and limiting its occupancy at inflammatory promoters, thus disrupting the positive feedback loop between activated immune cells and injured sensory neurons [148]. This inhibition is particularly important because persistent NF-κB activation sustains chronic cytokine production and contributes to prolonged peripheral sensitization.

Another important mechanism by which ATF3 regulates neuroimmune signaling involves modulation of the inflammasome [149]. The NLRP3 inflammasome is increasingly recognized as a major contributor to neuropathic pain through production of mature IL-1β and IL-18 [150]. Inflammasome activation is a tightly regulated process that occurs via two sequential molecular stages. The initial priming phase is triggered by TLR-mediated activation of NF-κB, which promotes the transcription of key inflammasome components, including NLRP3, pro-IL-1β, and pro-caspase-1 [151]. A subsequent activation signal, typically induced by extracellular ATP acting through P2X7 receptors, K^+^ efflux, mitochondrial ROS, lysosomal disruption, or intracellular Ca^2+^ overload, drives the assembly of the NLRP-ASC-caspase-1 inflammasome complex [152]. Once activated, caspase-1 processes the inactive precursors pro-IL-1β and pro-IL-18 into their biologically active forms [153].

Among these cytokines, IL-1β plays a central role in pain sensitization by enhancing glutamatergic neurotransmission, facilitating NMDA receptor phosphorylation, increasing VGSC activity, and ultimately promoting spinal neuronal hyperexcitability [154]. Current evidence indicates that ATF3 interferes with the priming stage of inflammasome activation by repressing NF-κB-dependent transcription of several inflammasome-related genes [148]. By repressing the expression of the inflammasome components, ATF3 limits IL-1β production and mitigates the propagation of pro-inflammatory signaling [155].

Neuroimmune signaling initiated in the PNS extends to the spinal dorsal horn, where activated microglia and astrocytes become major regulators of central sensitization [156]. Following peripheral nerve injury, primary afferent terminals release ATP, CSF1, CCL21, and CX3CL1, which activate microglial P2X4, P2X7, TLR4, and CX3CR1 receptors [157]. The activation of these receptors stimulates p38 MAPK signaling, inducing production of TNF-α, IL-1β, IL-6, prostaglandins, ROS, and BDNF [158,159]. BDNF released by activated microglia binds TrkB receptors on dorsal horn neurons, triggering downregulation of the K^+^-Cl^−^ cotransporter KCC2 [160]. Reduced KCC2 expression disrupts intracellular Cl^−^ homeostasis, weakening inhibitory GABAergic and glycinergic neurotransmission and producing neuronal disinhibition, a hallmark of central sensitization [161].

Finally, astrocytes also contribute markedly to chronic pain maintenance states [162]. Unlike microglia, which predominate during the early stages following injury, astrocytes become activated during chronic pain and sustain pro-inflammatory signaling long after the initial insult [163]. Pro-inflammatory cytokines, like IL-1β and TNF-α, activate STAT3 and NF-κB signaling in astrocytes, inducing expression of glial fibrillary acidic protein (GFAP), chemokines like CXCL1 and CCL2, matrix metalloproteinases (MMPs), and additional inflammatory mediators [164,165]. CXCL1 (produced by reactive astrocytes) activates neuronal CXCR2 receptors, enhancing excitatory neurotransmission and sustaining central sensitization [166]. Experimental evidence suggests that ATF3 may reduce reactive astrogliosis by antagonizing NF-κB and AP-1 activity, reducing cytokine production and limiting astrocyte-mediated amplification of spinal inflammation [167], although the underlying molecular mechanisms remain under active investigation.

### 3.4. ATF3 in Nociceptor Plasticity

ATF3 is a master regulator of the transcriptional reprogramming that occurs in nociceptors following peripheral nerve injury and is considered one of the earliest molecular signatures of the transition from physiological nociception to pathological pain [111]. In adult DRG neurons, ATF3 expression is virtually undetectable under basal conditions but is rapidly induced in damaged nociceptors subsequent to axotomy, mechanical trauma, chemotherapy, metabolic stress, or chronic inflammation [168]. Injury-dependent activation of MAPK signaling pathways, especially JNK, p38, and ERK, together with Ca^2+^ influx and ER stress, enables ATF3 transcription and nuclear accumulation [12,169]. Once activated, ATF3 acts as a stress-responsive transcription factor that heterodimerizes with AP-1 proteins to coordinate several changes in the nociceptor transcriptome, shifting these neurons from a homeostatic state toward an excitable and sensitized phenotype [41].

One of the major consequences of ATF3 activation is the remodeling of the molecular machinery controlling nociceptor excitability [170]. Whereas the induction of RAGs represents a regenerative transcriptional output of neuronal ATF3, the remodeling of ion-channel expression represents a distinct nociceptive output that can increase neuronal excitability [171]. ATF3 regulates transcriptional programs associated with membrane excitability, including increased expression of injury-associated VGSCs, particularly Nav1.3 [172]. In parallel, ATF3-associated transcriptional changes might reduce the expression of VGKCs, impairing membrane repolarization and lowering the threshold for action potential generation [173]. These modifications drive ectopic firing, repetitive discharge, and spontaneous activity in injured nociceptors, all of which are characteristic of the electrophysiological alterations underlying neuropathic pain [174]. ATF3 also contributes to the sensitization of polymodal nociceptors by modulating the expression and trafficking of transient receptor potential channels, like TRPV1 and TRPA1, increasing neuronal responsiveness to thermal, mechanical, and inflammatory stimuli [144].

ATF3 also profoundly influences the secretory phenotype of nociceptors, transforming them into active sources of inflammatory mediators that perpetuate peripheral sensitization. Injured ATF3-positive nociceptors exhibit increased production of neuropeptides such as CGRP and substance P, together with cytokines (e.g., IL-6) and chemokines (e.g., CCL2) [175]. These mediators recruit macrophages to the damaged nerve and DRG while promoting activation of satellite glial cells surrounding neuronal somata [176]. As a consequence, stimulated immune and glial cells release TNF-α, IL-1β, PGE_2_, ATP, NGF, and BDNF, all of which act on nociceptors to further increase Na^+^ channel activity, sensitize TRP channels, and activate intracellular signaling pathways such as ERK, PI3K/Akt, PKA, and PKC [177]. This reciprocal neuron-glia crosstalk establishes a self-perpetuating feed-forward loop that sustains nociceptor hyperexcitability long after the initial injury has resolved.

In addition to altering membrane excitability, ATF3 regulates the regenerative transcriptional program of nociceptors. It induces RAGs like *GAP43*, *SPRR1A*, galanin, *Sox11*, and *Jun*, which promote axonal growth and structural remodeling [102,103,104,178,179]. This regenerative transcriptional program represents an adaptive response to neuronal injury and is not, by itself, indicative of pathological pain. Although these responses are essential for peripheral nerve repair, they are accompanied by aberrant collateral sprouting and inappropriate reinnervation, processes that increase receptive field size and facilitate abnormal sensory input [180]. As a consequence, ATF3-dependent regeneration and ATF3-associated nociceptive plasticity can occur within the same injured neuron, with the final functional outcome depending on the balance between the transcriptional programs [98]. As a result, regenerated nociceptors usually display a hyperexcitable phenotype characterized by enhanced neurotransmitter release and persistent spontaneous activity, thus promoting long-lasting pain hypersensitivity [181].

Table 1 presents the most representative genes involved in pain associated with nerve injury that are directly or indirectly affected by the activity of ATF3.

Taken together, these findings suggest that the apparently opposing effects of ATF3 do not necessarily reflect contradictory functions of the transcription factor itself, but rather the activation of different downstream programs according to cellular context and injury state. Thus, ATF3 may promote regeneration when its activity is coupled to growth-associated and repair programs, while the same injury-induced factor may contribute to persistent pain when its activity is associated with sustained nociceptor excitability, aberrant sensory remodeling, and neuroimmune signaling (Table 2).

## 4. Experimental Evidence Across Pain Conditions

### 4.1. Neuropathic Pain

ATF3 has been widely investigated in experimental models of neuropathic pain and is consistently recognized as a key marker of neuronal injury and regeneration. Following ventral root avulsion (VRA) and spinal nerve transection (SNT), ATF3 is rapidly induced in injured DRG neurons and spinal motoneurons, with VRA producing a faster response than SNT, suggesting that ATF3 activation reflects the regenerative capacity of those injured neurons rather than pain itself [35]. In a model of DPN, ATF3 deficiency attenuated thermal hyperalgesia and mechanical allodynia, showing that ATF3 evokes neuropathic pain via the activation of p-PKCε and Bcl-XL in DRG neurons (Table 3) [36].

Beyond neurons, ATF3 also participates in immune responses linked to neuropathic pain. In the spared nerve injury (SNI) model, transcriptomic analyses identified DRG macrophages as the major immune-responsive cell population, in which ATF3 promoted JunB expression and the production of pro-inflammatory cytokines, indicating that ATF3 contributes to neuropathic pain by enhancing inflammatory signaling in macrophages [130]. Similarly, in a brachial plexus avulsion (BPA) model, ATF3 expression was markedly increased in uninjured DRG neurons adjacent to the lesion, suggesting that neuronal stress responses extend beyond directly injured neurons and might contribute to persistent mechanical hypersensitivity (Table 3) [169].

The relationship between ATF3 expression and pain persistence was supported in a lumbar radiculopathy (LR) model, where distal nerve constriction produced strong pain behaviors accompanied by a greater proportion of ATF3-positive DRG neurons, especially within NFH-positive sensory neurons, whereas proximal constriction induced greater hypoxia but reduced ATF3 expression. These findings suggest that ATF3 induction is associated with the development of persistent neuropathic pain [196]. Evidence from another DPN study demonstrated that ATF3, rather than CLEC5A, is a key mediator of ER stress, neuroinflammation, and oxidative imbalance, linking ATF3 activation to microglial activation, pro-inflammatory cytokine production, and reduced antioxidant defenses during DPN (Table 3) [197].

Beyond its role in neuropathic pain pathogenesis, ATF3 also allows peripheral nerve repair. Following facial nerve transection (FNT), loss of ATF3 impaired axonal regeneration and neurite outgrowth in adult DRG neurons. Transcriptomic analyses revealed that ATF3 positively regulates regeneration-associated neuropeptides, including VIP, galanin, PACAP, NGF, and GRP, while repressing inhibitory molecules such as CCL2. Moreover, administration of exogenous neuropeptides partially restored neurite growth in ATF3-deficient neurons, highlighting the role of ATF3 in coordinating transcriptional programs that support peripheral nerve regeneration (Table 3) [183].

This divergence suggests that ATF3 induction is not determined solely by the severity of tissue stress but also by the anatomical and cellular context of the injury, which may account for the absence of a uniform pain phenotype across models. Accordingly, ATF3 might exert context-dependent effects, promoting maladaptive inflammatory signaling in some neuropathic pain conditions while supporting adaptive neuronal responses and regeneration following nerve injury.

### 4.2. Inflammatory Pain

ATF3 is widely recognized as a critical marker of neuronal stress and injury and has also been implicated as a context-dependent modulator of pro-inflammatory signaling. Its expression has been characterized across many experimental models of acute and chronic inflammatory pain, revealing pronounced differences that depend on the cellular context and the nature of the inflammatory or nociceptive stimulus.

In models of acute inflammatory pain, ATF3 expression in DRG neurons varies according to the inflammatory stimulus. Carrageenan, capsaicin, formalin, mustard oil, and menthol increased ATF3 expression to different extents, with capsaicin- and mustard oil-induced effects being linked to TRPV1- and TRPA1-dependent mechanisms, respectively. In contrast, menthol-induced ATF3 upregulation occurred independently of TRPM8 activation, whereas CFA-induced chronic inflammation pain did not significantly affect ATF3 expression under these experimental conditions [168]. By contrast, CFA-induced monoarthritis markedly increased ATF3 expression in DRG neurons, predominantly in peptidergic neurons, with no association with pAKT signaling [198]. Similarly, i.pl. CFA induced sustained ATF3 expression, with ATF3-positive neurons co-expressing nociceptin and its receptor ORL1, underscoring a link between ATF3 expression and nociceptive signaling in the context of inflammation [193]. Additionally, in the CFA-induced pain model, 5-HT-deficient mice exhibited greater peripheral nerve fiber loss and increased ATF3 expression than wild-type mice, yet developed reduced thermal hyperalgesia; restoration of 5-HT restored the hyperalgesic phenotype [199]. This dissociation suggests that ATF3 may reflect neuronal injury or stress without necessarily predicting pain intensity.

In inflammatory joint diseases, ATF3 also plays distinct roles in neuronal and non-neuronal cells. In surgically induced osteoarthritis, IL-1β and TNF-α increased ATF3 expression in articular chondrocytes through NF-κB signaling, while ATF3 evoked IL-6 transcription and contributed to osteoarthritis progression [200]. In contrast, ATF3 induced an anti-inflammatory function in zymosan-induced peritonitis by repressing PTGS2/COX-2 transcription, thus limiting prostaglandin production and leukocyte accumulation [185]. In MIA-induced osteoarthritis, increased ATF3 and NPY expression in DRG neurons, together with GAP43 upregulation, further supported its association with sensory neuronal injury and regenerative responses [201]. Together, these effects suggest that the functional consequences of ATF3 activation are strongly dependent on the inflammatory context.

The main molecular mechanisms involving ATF3 across different experimental models of inflammatory pain are summarized in Table 4.

### 4.3. Cancer Pain

Cancer-associated pain is a pathological condition arising from tumor-induced alterations in peripheral sensory neurons, the tumor microenvironment, and central pain-processing pathways [202]. Some preclinical cancer pain models have demonstrated that tumor growth within or adjacent to tissues richly innervated by sensory afferents induces neuronal injury, neuroplasticity, and neuroimmune interactions that contribute to the development and maintenance of persistent pain. The main molecular mechanisms involving ATF3 across different experimental models of cancer pain are summarized in Table 5.

In bone cancer pain (BCP), injection of NCTC 2472 cells evokes tumor-associated injury of primary sensory afferent fibers innervating the bone. This neuronal damage is accompanied by increased expression of ATF3 and galanin in sensory neurons, molecular markers commonly associated with neuronal injury and phenotypic plasticity. In parallel, pronounced activation of satellite glial cells occurs within the ipsilateral DRG, characterized by glial fibrillary acidic protein (GFAP) upregulation and cellular hypertrophy. Macrophage infiltration in the ipsilateral DRG further indicates the involvement of neuroimmune mechanisms in the development of cancer-associated nociception [203].

A similar, but more complex, pattern of peripheral and central alterations has been described in a prostate cancer-induced bone pain model using RM1 cell injection. Tumor-bearing animals develop osteolytic lesions, aberrant bone formation, and reorganization of the local vascular network, macrophage populations, and sensory nerve fibers within the tumor-bearing bone. Moreover, increased ATF3 expression in ipsilateral DRG neurons reveals neuronal injury, whereas the concomitant upregulation of substance P and CGRP suggests enhanced nociceptive signaling. These peripheral alterations are accompanied by central sensitization and activation of glial cells within the ipsilateral spinal cord [204].

Cancer-associated pain is not restricted to bone malignancies. In head and neck cancer models, injection of OSCC cells induces sensory nerve injury and substantial neuronal plasticity. Tumor-associated sensory neurons show increased ATF3 expression, consistent with neuronal damage, together with increased neuronal excitability. Importantly, cancer-induced neural remodeling additionally involves the autonomic nervous system. Sympathetic postganglionic neurons undergo sprouting and transcriptional remodeling, resulting in enhanced norepinephrine release within the tumor-associated microenvironment. Injured ATF3-positive sensory neurons acquire increased expression or functional plasticity of α1-adrenergic receptors, rendering them more responsive to sympathetic-derived norepinephrine [205]. This illustrates the context-dependent consequences of ATF3-positive neuronal injury, which is linked to neuro-sympathetic rather than exclusively neuroimmune remodeling.

## 5. Conclusions

Accumulating evidence supports ATF3 as a central regulator of the cellular and molecular responses to neuronal injury and persistent pain. Although ATF3 was initially considered primarily a reliable marker of neuronal damage, current evidence indicates that its functions extend beyond injury detection. ATF3 integrates some stress-responsive signaling pathways, including the integrated stress response, MAPK, NF-κB, and unfolded protein response pathways, thus coordinating transcriptional programs involved in neuronal survival, axonal regeneration, inflammatory regulation, and nociceptor plasticity.

In the context of pain, ATF3 emerges as a multifaceted and context-dependent regulator. Its robust induction following peripheral nerve injury is consistently associated with neuronal stress and activation of regeneration-associated programs. In contrast, experimental evidence also indicates that ATF3 can contribute to persistent nociceptive sensitization via the regulation of neuronal excitability, neuropeptide expression, inflammatory signaling, and neuron–immune cell interactions. These distinct effects suggest that ATF3 should not be considered intrinsically pronociceptive or antinociceptive; rather, its functional consequences depend on the affected cell type, the nature and duration of the insult, the transcriptional partners involved, and the temporal profile of its activation.

An emerging body of evidence indicates that ATF3-mediated regulation of pain involves multiple cellular populations beyond injured neurons. Evidence from neuropathic, inflammatory, and cancer pain models indicates that ATF3 is expressed in and functionally relevant to several cellular populations, including macrophages, glial cells, Schwann cells, and other components of the injured PNS and CNS. By mediating these interactions, ATF3 may influence the balance between inflammatory amplification and resolution, thus modulating peripheral and central sensitization. Nevertheless, the direction and magnitude of these effects vary substantially between cellular contexts.

The available experimental evidence further suggests that ATF3 links neuronal injury to the transcriptional remodeling of nociceptors. Its activation is associated with changes in ion channel expression, neuropeptide signaling, regenerative gene programs, and neuron-glia communication, potentially establishing a feed-forward network that maintains nociceptor hyperexcitability after the initiating injury has subsided. At the same time, the induction of regeneration-associated genes indicates that some of these transcriptional responses may represent adaptive mechanisms that become maladaptive when sustained or improperly regulated. Thus, the temporal dynamics of ATF3 activation may be as important as its absolute expression level in determining whether its effects are predominantly reparative or contribute to chronic pain.

Evidence across neuropathic and inflammatory pain models reinforces the context-dependent nature of ATF3 signaling. In neuropathic pain, ATF3 has been associated with both peripheral nerve regeneration and the development or maintenance of pain-related phenotypes, including neuroinflammation and sensory hypersensitivity. In inflammatory pain, its induction is determined by the specific stimulus and tissue context, while its functional role may range from limiting inflammatory responses to promoting disease-associated cytokine production. Similarly, in cancer-associated pain, increased ATF3 expression is consistently associated with tumor-induced sensory neuronal injury and plasticity, but the consequences appear to involve interactions among sensory neurons, glial cells, immune populations, and the tumor microenvironment.

Importantly, a recent study provides a novel perspective by challenging the assumption that ATF3 induction directly drives pain. Following plantar incision, ATF3 was transiently induced in a subset of DRG neurons, but its deletion did not alter acute postoperative nociceptive behavior. Instead, transcriptomic analysis revealed a broader injury-responsive program, with the JNK-c-Jun pathway emerging as potential ATF3-independent regulators of postoperative hypersensitivity [206].

Future studies should clarify the cell-specific transcriptional programs governed by ATF3, define the molecular partners and chromatin-dependent mechanisms underlying ATF3-mediated transcriptional regulation, and determine how the duration of ATF3 activation influences the shift from adaptive repair to persistent nociceptive pathology. Particular attention should be given to the rare population of ATF3-positive neurons detected in apparently uninjured DRGs in both mice and humans, to determine whether these cells represent a distinct neuronal subtype or a transient regenerative state [207]. In light of the previously aforementioned findings, it will be very important to determine whether ATF3 functions primarily as a marker or modulator of neuronal injury in specific contexts, and to identify the alternative transcriptional pathways that mediate nociceptor plasticity and pain following different types of injury. Moreover, future studies integrating single-cell and spatial transcriptomics, epigenomic profiling, and cell-specific genetic manipulation should help resolve these questions and distinguish the beneficial regenerative functions of ATF3 from those that contribute to chronic pain.

From a translational perspective, the functional complexity and context dependency of ATF3 highlight the importance of selectively modulating its activity rather than pursuing broad systemic intervention. Therapeutic strategies targeting ATF3-dependent pathways will require cell-specific, pathway-specific, and controlled approaches that preserve its regenerative and homeostatic functions while limiting maladaptive inflammatory and nociceptive signaling. A more precise understanding of ATF3 as a dynamic transcriptional regulator, rather than merely a biomarker of neuronal injury, may therefore provide new opportunities for the development of mechanism-based interventions for chronic pain.

## Figures and Tables

**Table 1:** Evidence supporting ATF3-dependent regulation of key genes involved in pain neurotransmission. Abbreviations: Nav1.3 (voltage-gated sodium channel Nav1.3 -SCN3A-); TRPV1 (transient receptor potential vanilloid 1); TRPA1 (transient receptor potential ankyrin 1); TRPM8 (transient receptor potential melastatin 8); GAP43 (growth-associated protein 43); SPRR1A (small proline-rich protein 1A); NGF (nerve growth factor); GDNF (glial cell line-derived neurotrophic factor); VIP (vasoactive intestinal peptide); PACAP (pituitary adenylate cyclase-activating polypeptide); NPY (neuropeptide Y); CCL2 (C-C motif chemokine ligand 2); COX-2 (cyclooxygenase-2); PTGS2 (prostaglandin-endoperoxide synthase 2); TNF-α (tumor necrosis factor-alpha); TLR4 (Toll-like receptor 4); IL-6 (interleukin-6); IL-12 (interleukin-12); NLRP3 (NOD-, LRR-, and pyrin domain-containing protein 3); IL-1β (interleukin 1 beta); IL-18 (interleukin 18); Bcl-XL (B-cell lymphoma-extra large); PKCε (protein kinase C epsilon); ORL1 (opioid receptor-like 1); CLEC5A (C-type lectin domain family 5 member A); HSP27 (heat shock protein 27); ARG1 (arginase 1); GFRα1 (glial cell line-derived neurotrophic factor family receptor alpha 1); KCC2 (potassium-chloride cotransporter 2); CXCL1 (C-X-C motif chemokine ligand 1); N/A (not applicable).

Gene	Biological Process	Evidence of ATF3 Regulation	References
Nav1.3 (SCN3A)	Neuronal excitability	Indirect	[172]
TRPV1	Nociceptor sensitization	Unknown	N/A
TRPA1	Nociceptor sensitization	Unknown	N/A
TRPM8	Nociceptor sensitization	Unknown	N/A
GAP43	Axonal regeneration	Indirect	[94]
SPRR1A	Axonal regeneration	Direct	[20]
NGF	Axonal regeneration Neurotrophic signaling	Indirect	[108]
GDNF	Axonal regeneration Neurotrophic signaling	Indirect	[108]
Galanin	Axonal regeneration Neuropeptide signaling	Indirect	[182,183]
VIP	Axonal regeneration Neuropeptide signaling	Indirect	[183]
PACAP	Axonal regeneration Neuropeptide signaling	Indirect	[183]
NPY	Axonal regeneration Neuropeptide signaling	Direct	[184]
CCL2	Neuroimmune signaling	Indirect	[175,183]
COX-2	Inflammation	Direct	[185]
PTGS2	Inflammation	Direct	[185]
TNF-α	Inflammation	Direct	[186]
TLR4	Inflammation	Direct	[187]
IL-6	Inflammation	Direct	[188]
IL-12	Inflammation	Direct	[189]
NLRP3	Inflammasome activation	Indirect	[190]
IL-1β	Inflammasome activation Inflammation	Indirect	[40]
IL-18	Inflammasome activation Inflammation	Direct	[191]
Bcl-XL	Neuronal survival	Direct	[36,192]
PKCε	Nociceptive signaling	Direct	[36]
ORL1	Nociceptive signaling	Unknown	[193]
CLEC5A	Neuroinflammation	Unknown	N/A
HSP27	Regeneration Stress response	Direct	[96]
ARG1	Axonal regeneration	Indirect	[194]
GFRα1	Neurotrophic signaling	Indirect	[107]
KCC2	Central sensitization	Unknown	N/A
CXCL1	Astrocyte-mediated sensitization	Direct	[195]

**Table 2:** Functional roles and outcomes of ATF3 across neuronal and non-neuronal cells in peripheral nerve regeneration. Abbreviations: ATF3 (activating transcription factor 3).

Cell Type	ATF3 Context	Predominant ATF3 Role	Functional Outcome	References
Peripheral neurons	Induced after peripheral nerve injury	Promotes a regenerative transcriptional response, supporting neuronal survival, and axonal remodeling	Axonal regeneration and functional repair	[183]
Nociceptive neurons	Induced after nerve injury and maintained during neuronal stress	Coordinates regenerative responses with changes in neuronal excitability and sensory plasticity	Regeneration accompanied by nociceptor sensitization and pain	[168]
Macrophages and other innate immune cells	Induced in response to inflammatory and injury-related signals	Acts predominantly as a transcriptional regulator that limits pro-inflammatory signaling	Modulation of the neuroimmune environment	[72]
Non-neuronal cells of the injured nerve	Induced as part of the local injury response	Contributes to regulation of inflammatory pathways, thereby influencing the environment surrounding injured neurons	Modulation of the regenerative microenvironment	[21]

**Table 3:** Summary of the molecular mechanisms involving ATF3 in different experimental models of neuropathic pain. This table summarizes the neuropathic pain models, the main cellular sources in which ATF3 expression was identified, and the associated molecular mechanisms. Abbreviations: ATF3 (activating transcription factor 3); VRA (ventral root avulsion); SNT (spinal nerve transection); DRG (dorsal root ganglion); DPN (diabetic peripheral neuropathy); WT (wild type); p-PKCε (phosphorylated protein kinase C epsilon); Bcl-XL (B-cell lymphoma-extra large); SNI (spared nerve injury); scRNA seq (single-cell RNA sequencing); JunB (JunB transcription factor); BPA (brachial plexus avulsion); LR (lumbar radiculopathy); NFH (neurofilament heavy chain); CGRP (calcitonin gene-related peptide); KO (knockout); ER stress (endoplasmic reticulum stress); FNT (facial nerve transection); VIP (vasoactive intestinal peptide); PACAP (pituitary adenylate cyclase-activating polypeptide); NGF (nerve growth factor); GRP (gastrin-releasing peptide); and CCL2 (C-C motif chemokine ligand 2).

Neuropathic Pain Model	Species Tested	Cellular Source of ATF3	Molecular Mechanisms	References
VRA SNT	Female Sprague-Dawley rats	Injured DRG neurons Spinal motoneurons	ATF3 is a stress-induced marker that is upregulated in sensory and motor neurons following VRA and SNT neuropathic pain models. Following SNT, ATF3 immunoreactivity appeared in DRG neurons within 6 h and in spinal motoneurons after 24 h. In contrast, VRA induced a much faster response, with most motoneurons expressing ATF3 within 3–6 h	[35]
DPN	ATF3^+/+^ and ATF^−/−^ mice (C57BL/6 background)	Injured DRG neurons	This study examined the role of ATF3 in DPN using WT and ATF3^−/−^ mice. While both groups developed hyperglycemia and comparable skin denervation, only WT mice exhibited thermal hyperalgesia and mechanical allodynia. ATF3 upregulation was accompanied by increased expression of p-PKCε and the protein Bcl-XL in DRG neurons, whereas these responses were markedly attenuated in ATF3^−/−^ mice	[36]
SNI	Male mice (strain not reported)	DRG macrophages	This study investigated the role of immune-related genes in the DRG during neuropathic pain using bulk and scRNA-seq in a SNI model. Macrophages showed the highest immune-related gene activity, and ATF3 and JunB were identified as key transcription factors associated with inflammatory responses. In vitro, ATF3 upregulation promoted JunB expression and the release of pro-inflammatory cytokines, whereas ATF3^−/−^ reduced both JunB expression and cytokine production	[130]
BPA	Male Wistar rats	Injured DRG neurons	ATF3 expression was evaluated in a rat model of BPA to investigate its relationship with neuropathic pain. Rats subjected to C8–T1 root avulsion developed persistent mechanical hyperalgesia in dermatomes innervated by uninjured nerves for up to 21 days. This fact was accompanied by a significant increase in ATF3 immunoreactivity in DRG neurons at the C5–C7 levels	[169]
FNT	ATF3^+/+^ and ATF^−/−^ mice (C57BL/6 background)	Injured DRG neurons	ATF3 plays a role in peripheral nerve regeneration, as its loss impairs facial nerve regeneration and reduces neurite outgrowth in DRG neurons. Transcriptomic analyses identified a cluster of regeneration- associated neuropeptide genes, including VIP, Galanin, PACAP, NGF, and GRP, that are positively regulated by ATF3, while growth-inhibitory molecules such as CCL2 are suppressed. Moreover, administration of exogenous neuropeptides partially restored neurite growth in ATF3-deficient neurons	[183]
LR	Male Sprague-Dawley rats	Injured DRG neurons	Nerve constriction distal to the DRG produced more persistent pain behaviors than constriction proximal to the DRG. This was associated with a significantly higher proportion of ATF3-positive DRG neurons, particularly within NFH-positive neurons, whereas both injury models increased ATF3 expression in CGRP-positive neurons	[196]
DPN	ATF3^+/+^ and CLEC5A^+/+^ ATF^−/−^ and CLEC5A^−/−^ mice (C57BL/6 background)	Injured DRG neurons	This study investigated the key roles of ATF3 and CLEC5A in the development of DPN using KO mice. Diabetic WT and CLEC5A^−/−^ mice developed neuropathic pain, ER stress, neuroinflammation, and increased ATF3 expression, whereas these changes were absent in ATF3^−/−^ mice. ATF3 upregulation was associated with activation of ER stress pathways, microglial activation, pro-inflammatory cytokine release, and reduced expression of several antioxidant enzymes	[197]

**Table 4:** Summary of the molecular mechanisms involving ATF3 in distinct experimental models of inflammatory pain. This table summarizes the inflammatory pain models, the main cellular sources in which ATF3 expression was identified, and the associated molecular mechanisms. Abbreviations: CFA (complete Freund’s adjuvant); DRG (dorsal root ganglion); ATF3 (activating transcription factor 3); TRPV1 (transient receptor potential vanilloid 1); TRPA1 (transient receptor potential ankyrin 1); TRPM8 (transient receptor potential melastatin 8); Mrgprd^DTR^ (transgenic mice expressing diphtheria toxin receptor in Mrgprd+ neurons); pAKT (phosphorylated AKT); IL-1β (interleukin-1 beta); TNF-α (tumor necrosis factor alpha); NF-κB (nuclear factor kappa-light-chain-enhancer of activated B cells); IL-6 (interleukin 6); IL-8 (interleukin 8); PTGS2 (prostaglandin-endoperoxide synthase 2); COX-2 (cyclooxygenase-2); PGE_2_ (prostaglandin E_2_); PGD_2_ (prostaglandin D_2_); MIA (monosodium iodoacetate); NPY (neuropeptide Y); GAP43 (growth associated protein 43); I.pl. (intraplantar injection); ORL1 (opioid receptor-like 1); 5-HT (5-hydroxytryptamine -serotonin-); and WT (wild type).

Inflammatory Pain Model	Species Tested	Cellular Source of ATF3	Molecular Mechanisms	References
Acute inflammatory pain (carrageenan, capsaicin, formalin, mustard oil, and menthol injection) Chronic inflammatory pain (CFA injection)	TRPV1-, TRPA1-, and TRPM8-null mice Mrgprd^DTR^ mice (strain not reported)	DRG neurons	CFA did not increase ATF3 expression compared with needle-injected controls, whereas carrageenan produced a small increase in ATF3-positive DRG neurons. Capsaicin induced a dose-dependent increase in ATF3 expression in DRG neurons in a TRPV1-dependent manner. Formalin produced an increase in ATF3 expression, while mustard oil similarly induced ATF3 expression, with these effects being associated with TRPA1 activation at lower doses. Menthol increased the number of ATF3-positive DRG neurons, although this effect was independent of TRPM8 activation	[168]
Peritonitis (zymosan injection)	ATF3^+/+^ and ATF^−/−^ mice (C57BL/6J background)	Peritoneal leukocytes	Zymosan stimulation induced a transient increase in ATF3 expression together with PTGS2/COX-2. ATF3 was recruited to the PTGS2 promoter, where it acted as a transcriptional repressor. ATF3-deficient mice showed increased COX-2 expression, higher PGE_2_ and PGD_2_ production, and greater leukocyte accumulation during acute peritonitis	[185]
Chronic inflammatory pain (CFA injection)	Male Sprague-Dawley rats	DRG neurons	I.pl. CFA induced ATF3 expression in DRG neurons at 7 days, with persistence at 14 days. ATF3-positive neurons also expressed nociceptin and its receptor ORL1	[193]
Monoarthritis (CFA injection)	Male Wistar rats	DRG neurons	Monoarthritis produced a marked increase in ATF3 expression, indicating neuronal injury or stress in primary afferents during inflammatory joint pain. ATF3-positive neurons were mainly peptidergic and showed no significant association with pAKT	[198]
Chronic inflammatory pain (CFA injection)	5-HT^+/+^ and 5-HT^/−^ mice (C57BL/6J background)	DRG neurons	CFA-induced inflammation caused loss of nerve fibers and a higher number of ATF3-positive DRG neurons in 5-HT^−/−^ mice than in WT animals. Despite this increased peripheral nerve injury, 5-HT^−/−^ mice developed reduced thermal hyperalgesia because of lower serotonin levels. Administration of 5-HT restored the hyperalgesic phenotype, indicating that 5-HT and ATF3 modulates inflammatory pain	[199]
Osteoarthritis (surgery)	Col2a1-Cre; Atf3^fl/fl^ mice (strain not reported)	Articular chondrocytes	Several pro-inflammatory cytokines (IL-1β and TNF-α) increased ATF3 expression through NF-κB signaling, while ATF3 promoted IL-6 transcription. ATF3^−/−^ reduced NF-κB activation and attenuated IL-6 production and osteoarthritis development	[200]
Osteoarthritis (MIA injection)	Male Wistar rats	DRG neurons	Expression of the neuronal injury markers ATF3 and NPY, together with the regeneration-associated protein GAP43, was assessed in DRG following intra-articular administration of MIA at multiple time points. Osteoarthritis induction produced characteristic joint pathology and nociceptive behaviors, accompanied by an early increase in ATF3 and NPY expression and a marked upregulation of GAP43 in ATF3-positive neurons	[201]

**Table 5:** Summary of the molecular mechanisms involving ATF3 in distinct experimental models of cancer pain. This table summarizes the cancer pain models, the main cellular sources in which ATF3 expression was identified, and the mechanistic basis. Abbreviations: BCP (bone cancer pain); NCTC (National Collection of Type Cultures); DRG (dorsal root ganglion); ATF3 (activating transcription factor 3); GFAP (glial fibrillary acidic protein); RM1 (Ras/Myc-Cap 1 cell line); CGRP (calcitonin gene-related peptide); and OSCC (oral squamous cell carcinoma).

Cancer Pain Model	Species Tested	Cellular Source of ATF3	Molecular Mechanisms	References
BCP (NCTC 2472 cells injection)	Male C3H/HeJ mice	Injured DRG neurons	Tumor-induced injury of primary sensory afferent fibers innervating the bone is associated with increased ATF3 and galanin expression, together with satellite glial cell activation, characterized by GFAP upregulation and macrophage infiltration in the ipsilateral DRG	[203]
BCP (RM1 cells injection)	Male C57BL/6 mice	Injured DRG neurons	Prostate cancer-induced bone pain was associated with progressive pain, accompanied by osteolytic lesions, aberrant bone formation, and reorganization of blood vessels, and sensory nerve fibers within the tumor-bearing bone. At the neuronal level, increased ATF3 expression together with upregulation of substance P and CGRP in ipsilateral DRG neurons indicated neuronal injury and enhanced nociceptive signaling. These peripheral changes were accompanied by central sensitization and glial activation in the spinal cord	[204]
Head and neck cancer (OSCC cells administration)	Male and female C57BL/6 mice	Sensory nerve injured	OSCC induces sensory nerve injury, evidenced by the upregulation of ATF3 in sensory neurons. In parallel, sympathetic postganglionic neurons undergo sprouting, transcriptional remodeling, and hyperexcitability, leading to increased norepinephrine release. Injured ATF3^+^ sensory neurons acquire α1-adrenergic receptor plasticity, becoming more responsive to norepinephrine	[205]

## Data Availability

No new data were created or analyzed in this study. Data sharing is not applicable to this article.

## Abbreviations

The following abbreviations are used in this manuscript:

5-HT	5-hydroxytryptamine (serotonin)
Akt/PKB	Protein kinase B
AMPK	AMP-activated protein kinase
AP-1	Activator protein 1
Arg1	Arginase 1
ASC	Apoptosis-associated speck-like protein containing a CARD
ASK1	Apoptosis signal-regulating kinase 1
ASIC	Acid-sensing ion channel
ATF	Activating transcription factor
ATF2	Activating transcription factor 2
ATF3	Activating transcription factor 3
ATF4	Activating transcription factor 4
ATF6	Activating transcription factor 6
ATM	Ataxia telangiectasia mutated
ATP	Adenosine triphosphate
ATR	ATM and Rad3-related
Bcl-XL	B-cell lymphoma-extra large
BCP	Bone cancer pain
BDNF	Brain-derived neurotrophic factor
BPA	Brachial plexus avulsion
bZIP	Basic leucine zipper
C5–C7	Cervical spinal segments 5–7
C8–T1	Cervical 8-thoracic 1 spinal roots
Ca^2+^	Calcium ion
C/EBP	CCAAT/enhancer binding protein
c-Jun	Transcription factor Jun
CaMK	Ca^2+^/calmodulin-dependent protein kinases
cAMP	Cyclic adenosine monophosphate
CAP23	Cytoskeleton-associated protein 23
CBP	CREB-binding protein
CCI	Chronic constriction injury
CCL2	C-C motif chemokine ligand 2
CCL4	C-C motif chemokine ligand 4
CCL21	C-C motif chemokine ligand 21
CCR2	C-C Motif chemokine receptor 2
Cdc42	Cell division cycle 42
CFA	Complete Freund’s adjuvant
CGRP	Calcitonin gene-related peptide
CHOP	C/EBP homologous protein
CIPN	Chemotherapy-induced peripheral neuropathy
Cl^−^	Chloride ion
CLEC5A	C-type lectin domain family 5 member A
CNS	Central nervous system
COX-2	Cyclooxygenase-2
CRE	cAMP response element
CREB	cAMP response element-binding protein
CSF1	Colony-stimulating factor 1
CSF1R	Colony-stimulating factor 1 receptor
CSPG	Chondroitin sulfate proteoglycan
CX3CL1	C-X3-C Motif chemokine ligand 1
CX3CR1	C-X3-C Motif chemokine receptor 1
CXCL1	C-X-C Motif chemokine ligand 1
CXCR2	C-X-C Motif chemokine receptor 2
DAMP	Damage-associated molecular pattern
DDIT3	DNA damage inducible transcript 3
DNA	Deoxyribonucleic acid
DPN	Diabetic peripheral neuropathy
DRG	Dorsal root ganglion
eIF2α	Eukaryotic translation initiation factor 2 alpha
EIF2AK1	Eukaryotic translation initiation factor 2 alpha kinase 1
EIF2AK2	Eukaryotic translation initiation factor 2 alpha kinase 2
EIF2AK3	Eukaryotic translation initiation factor 2 alpha kinase 3
EIF2AK4	Eukaryotic translation initiation factor 2 alpha kinase 4
ELAVL1	ELAV-like RNA binding protein 1
ER	Endoplasmic reticulum
ERK	Extracellular signal-regulated kinase
FNT	Facial nerve transection
GAP43	Growth-associated protein 43
GCN2	General control nonderepressible 2
GDNF	Glial cell line-derived neurotrophic factor
GFAP	Glial fibrillary acidic protein
GFRα1	GDNF family receptor alpha 1
GM-CSF	Granulocyte-macrophage colony-stimulating factor
GRP	Gastrin-releasing peptide
GTPase	Guanosine triphosphatase
H3	Histone H3
H3K4me3	Histone H3 lysine 4 trimethylation
H3K27me3	Histone H3 lysine 27 trimethylation
H4	Histone H4
HDAC	Histone deacetylase
HDAC1	Histone deacetylase 1
HDAC2	Histone deacetylase 2
HMGB1	High mobility group box 1
HRI	Heme-regulated inhibitor
HSP27	Heat shock protein 27
HuR	Human antigen R
IASP	International association for the study of pain
IκB	Inhibitor of nuclear factor kappa B
IKK	IκB kinase
IKKα	IκB kinase alpha
IKKβ	IκB kinase beta
IKKγ	IκB kinase gamma
IRE1α	Inositol-requiring enzyme 1 alpha
IL-1β	Interleukin 1 beta
IL-6	Interleukin 6
IL-8	Interleukin 8
IL-12	Interleukin 12
IL-18	Interleukin 18
I.pl.	Intraplantar injection
IRAK1	Interleukin-1 receptor-associated kinase 1
IRAK2	Interleukin-1 receptor-associated kinase 2
IRAK4	Interleukin-1 receptor-associated kinase 4
ISR	Integrated stress response
JNK	c-Jun N-terminal kinase
JunB	JunB transcription factor
K^+^	Potassium ion
KCC2	Potassium-chloride cotransporter 2
kDa	Kilodalton
KLF	Krüppel-like factor
KLF4	Krüppel-like factor 4
KLF6	Krüppel-like factor 6
KLF7	Krüppel-like factor 7
KO	Knockout
LR	Lumbar radiculopathy
MAPK	Mitogen-activated protein kinase
MDM2	Mouse double minute 2
Mrgprd^DTR^	Transgenic mice expressing diphtheria toxin receptor in Mrgprd+ neurons
MKK3	Mitogen-activated protein kinase kinase 3
MKK4	Mitogen-activated protein kinase kinase 4
MKK6	Mitogen-activated protein kinase kinase 6
MKK7	Mitogen-activated protein kinase kinase 7
MIA	Monosodium iodoacetate
miR-27a-3p	microRNA-27a-3p
miR-488	microRNA 488
miR-494	microRNA 494
MMP	Matrix metalloproteinase
mRNA	Messenger RNA
mTOR	Mechanistic target of rapamycin
MyD88	Myeloid differentiation primary response protein 88
N/A	Not applicable
Na^+^	Sodium ion
Nav1.3	Voltage-gated sodium channel Nav1.3 (SCN3A)
Nav1.7	Voltage-gated sodium channel Nav1.7 (SCN9A)
Nav1.8	Voltage-gated sodium channel Nav1.8 (SCN10A)
Nav1.9	Voltage-gated sodium channel Nav1.9 (SCN11A)
NCoR	Nuclear receptor co-repressor
NCTC	National Collection of Type Cultures
NF-κB	Nuclear factor kappa-light-chain-enhancer of activated B cells
NFH	Neurofilament heavy chain
NGF	Nerve growth factor
NLRP3	NLR family pyrin domain containing 3
NMDA	N-methyl-D-aspartate
NO	Nitric oxide
NPY	Neuropeptide Y
ORL1	Opioid receptor-like 1
OSCC	Oral squamous cell carcinoma
p-PKCε	Phosphorylated protein kinase C epsilon
PKCε	Protein kinase C epsilon
P2X3	Purinergic receptor P2X3
P2X4	Purinergic receptor P2X4
P2X7	Purinergic receptor P2X7
p300	E1A-binding protein p300 (EP300)
p38 MAPK	p38 mitogen-activated protein kinase
p53	Tumor protein p53
PACAP	Pituitary adenylate cyclase-activating polypeptide
pAKT	Phosphorylated AKT
PERK	PKR-like endoplasmic reticulum kinase
PGD_2_	Prostaglandin D_2_
PGE_2_	Prostaglandin E_2_
PI3K	Phosphoinositide 3-kinase
PKA	Protein kinase A
PKC	Protein kinase C
PKR	Protein kinase R
PNS	Peripheral nervous system
pro-IL-1β	Pro-interleukin-1 beta
pro-IL-18	Pro-interleukin-18
PTEN	Phosphatase and tensin homolog
PTGS2	Prostaglandin-endoperoxide synthase 2
Rac1	Rac family small GTPase 1
RAG	Regeneration-associated gene
RAGE	Receptor for advanced glycation end product
RATF	Regeneration-associated transcription factor
RhoA	Ras homolog family member A
RM1	Ras/Myc-Cap 1 cell line
RNA	Ribonucleic acid
ROS	Reactive oxygen specie
SCI	Spinal cord injury
scRNA-seq	Single-cell RNA sequencing
Ser63	Serine 63
Ser73	Serine 73
SIN3A	SIN3 transcription regulator family member A
Smad1	SMAD family member 1
SMRT	Silencing mediator for retinoid and thyroid hormone receptor
SNI	Spared nerve injury
SNL	Spinal nerve ligation
SNT	Sciatic nerve transection
Sox11	SRY-Box transcription factor 11
SPRR1A	Small proline-rich protein 1A
STAT3	Signal transducer and activator of transcription 3
SUMO	Small ubiquitin-like modifier
TAK1	Transforming growth factor-β-activated kinase 1
TAK2	Transforming growth factor-β-activated kinase 2
TAK3	Transforming growth factor-β-activated kinase 3
TBI	Traumatic brain injury
TIRAP	Toll/interleukin-1 receptor domain-containing adaptor protein
TNF-α	Tumor necrosis factor alpha
TLR	Toll-like receptor
TLR4	Toll-like receptor 4
TRAF6	Tumor necrosis factor receptor-associated factor 6
TRIF	TIR-domain-containing adapter-inducing interferon-β
TrkB	Tropomyosin receptor kinase B
TRP	Transient receptor potential channel
TRPA1	Transient receptor potential ankyrin 1
TRPM8	Transient receptor potential melastatin 8
TRPV1	Transient receptor potential vanilloid 1
UPR	Unfolded protein response
UTR	Untranslated region
VIP	Vasoactive intestinal peptide
VGCC	Voltage-gated calcium channel
VGKC	Voltage-gated potassium channel
VGSC	Voltage-gated sodium channel
VRA	Ventral root avulsion
WT	Wild type
XBP1	X-box binding protein 1
